# Optimization of stimulus color for peripheral SSVEP-based brain-computer interfaces

**DOI:** 10.3389/fnhum.2026.1832475

**Published:** 2026-06-30

**Authors:** Haochen Liu, Zhaohui Li, Wenwen Li, Ruoqi Yang, Xiaogang Chen

**Affiliations:** 1 Institute of Biomedical Engineering, Chinese Academy of Medical Sciences and Peking Union Medical College, Tianjin, China; 2 School of Biomedical Engineering and Technology, Tianjin Medical University, Tianjin, China; 3 CASIC-CQC Software Testing and Assessment Technology (Beijing) Co., Ltd., Beijing, China; 4 School of Optometry and Ophthalmology, Tianjin Medical University, Tianjin, China

**Keywords:** brain-computer interfaces, color, electroencephalogram, peripheral vision field, steady-state visual evoked potentials

## Abstract

**Background:**

Most existing steady-state visual evoked potential (SSVEP)-based brain-computer interfaces (BCIs) struggle to balance user experience with system performance. Although recent studies have shown that peripheral vision stimulation can evoke SSVEPs with high user comfort, the impact of stimulus color on peripheral SSVEP performance remains underexplored. Therefore, this study attempted to investigate the effect of stimulus color on peripheral SSVEPs.

**Methods:**

Four conventional stimulus colors (i.e., blue, green, red, and white) were evaluated using ultra-low frequency SSVEP stimuli, with the stimulation frequencies ranging from 2 Hz to 3.32 Hz. Based on the results, the optimized stimulus color was used to build a 12-target peripheral SSVEP-based BCI. Task-discriminant component analysis (TDCA) algorithm was adopted to detect SSVEPs. The feasibility of the proposed system was verified through offline experiments with 13 participants and online experiments with 11 participants.

**Results:**

The offline experiments with 13 participants showed no significant differences in classification accuracy and information transfer rates (ITRs) among the four-color paradigms. However, green stimulation received the highest subjective comfort ratings. Consequently, green stimulation was selected for building the 12-target peripheral SSVEP-based BCI. The online results achieved a mean classification accuracy of 89.93 ± 6.10% and an ITR of 47.96 ± 6.98 bits/min.

**Conclusion:**

The present findings support a comfort-driven color selection strategy for peripheral ultra-low-frequency SSVEP stimulation while maintaining comparable performance among the tested colors. These findings may provide practical guidance for more visually tolerable SSVEP-based BCI systems based on peripheral visual stimulation.

## 1 Introduction

Brain-computer interface (BCI) is a communication and control system without relying on the brain’s normal neural and muscle output pathways (Wolpaw et al., 2002; Pfurtscheller et al., 2017; Gao et al., 2021). Over the past decade, BCI has demonstrated widespread potential in medical rehabilitation (Zhang et al., 2017; Baniqued et al., 2021), communication assistance (Chen et al., 2022; Zhang R. et al., 2023), and intelligent control (Chen et al., 2019; Zhang S. et al., 2023; Kim et al., 2025). Among the existing BCI paradigms, steady-state visual evoked potential (SSVEP)-based BCI has attracted much attention due to its high information transfer rates (ITRs) (Li et al., 2024; Ke et al., 2026), strong signal stability over time, and relatively low user-training barriers compared to active BCI paradigms (Chen et al., 2021).

The operation mechanism of the SSVEP-based BCI system relies on external visual stimuli to induce specific neural oscillatory responses in the occipital cortex, which requires an efficient and visually tolerable visual stimulation interface. Therefore, the design of stimulation parameters directly impacts signal quality and user experience (Reitelbach and Oyibo, 2024). Among a variety of influencing factors, stimulus color plays an important role due to its unique visual physiological properties. Conventional SSVEP stimuli often rely on flickering stimuli with strong luminance modulation, which can induce visual discomfort and fatigue during prolonged use (Xie et al., 2016; Ladouce et al., 2022). To address this issue, chromatic stimulation has been explored as an alternative. Stimulus color can modulate SSVEP responses which may be related to the engagement of visual pathways originating from cone photoreceptors with different spectral sensitivities (Schnapf et al., 1987; Tello et al., 2015). Although chromatic stimulation holds the potential to improve user comfort (Azadi Moghadam and Maleki, 2023; Kim et al., 2025), selecting the optimal stimulus color in conventional paradigms often presents a dilemma. For instance, Tello et al. (2015) reported that red stimuli produced a higher ITR than other tested colors, but were rated as the least comfortable and could even raise safety concerns. Similarly, Du and Zhao (2022) found that red stimuli achieved the highest ITR in PC-based SSVEP, whereas blue stimulation showed the poorest performance in both AR- and PC-based SSVEP systems. Regarding user experience, however, green and blue stimuli have often been regarded as more visually tolerable (Reitelbach and Oyibo, 2024). These findings suggest a clear trade-off between SSVEP performance and user comfort across different stimulus colors. Consequently, in most cases, white remains the conventional default choice (Zhu et al., 2010). It is important to note that all the abovementioned studies used central visual field stimulation to induce SSVEPs. It remains unknown whether peripheral visual stimulation has a similar color-dependent effect on SSVEPs.

Recent studies have proposed several comfort-oriented visual BCI strategies to reduce visual discomfort while maintaining system performance. These strategies include reduced modulation depth or high-frequency SSVEP stimuli (Ladouce et al., 2022), non-binary gray-level c-VEP sequences (Martínez-Cagigal et al., 2023), low-pixel-density or subtle-flicker SSVEP stimuli (Meng et al., 2023; Ming et al., 2023b), and textured c-VEP stimuli (Dehais et al., 2024). In parallel, stimulation delivered to the peripheral visual field can also elicit reliable SSVEPs, allowing users to avert their gaze from the flicker source, thereby improving comfort and reducing central vision occlusion (Lee et al., 2018). For instance, Zhao et al. (2021) compared the conventional SSVEP with a peripheral SSVEP paradigm. Their results indicated significantly higher comfort ratings for the peripheral stimulation than for conventional SSVEP, confirming its efficacy in mitigating visual fatigue. Similarly, Hu et al. (2023) investigated sub-regions of the peripheral annular visual field by using a four-class spatially-coded BCI paradigm. They achieved a high-performance peripheral SSVEP-based BCI, with an online information transfer rate (ITR) of 22.48 ± 6.71 bits/min and an online classification accuracy of 87.50 ± 9.13%.

Although peripheral SSVEP paradigms have demonstrated advantages in user comfort and gaze flexibility, the existing studies have predominantly relied on white flicker stimuli (Duart et al., 2020), with limited exploration of color’s role in peripheral vision. Given that distinct cone distributions in the central versus peripheral retina led to differential color processing, which may affect SSVEP responses, investigating chromatic stimuli in peripheral vision is therefore essential (Liu et al., 2024). To address this issue, the present study combined color stimulation with ultra-low frequency modulation in a peripheral SSVEP-based BCI. While both system performance and user experience were evaluated, the research focused on a comfort-driven color selection within this laboratory paradigm. This study aimed to explore the impact of stimulus color on BCI system performance in peripheral vision filed and provide a foundation for subsequent construction of a more visually tolerable system.

## 2 Experimental procedures

### 2.1 Participants

The experiment involved 15 participants (7 females and 8 males, aged 18–25 years, mean age 20.6 years). All participants were university students or researchers with prior experience in BCI experiments, ensuring their high reliability and strict adherence to experimental instructions. A total of 13 participants engaged in offline experiment while 11 participated in online experiment. Among them, 9 participants carried out both offline and online experiments. The fact that some participants did not take part in both experiments was primarily due to graduation and unavoidable scheduling conflicts during the data collection period. All participants read and signed an informed consent form approved by the Institutional Review Board of Tsinghua University before the experiment. The study was conducted in accordance with the Declaration of Helsinki, and all ethical and experimental procedures were approved by the Ethics Review Committee for Life Science and Medical Research Involving Humans, Institute of Biomedical Engineering, Chinese Academy of Medical Sciences under Application No. L2025-045-001.

### 2.2 Visual stimulus presentation

**Figure 1:**
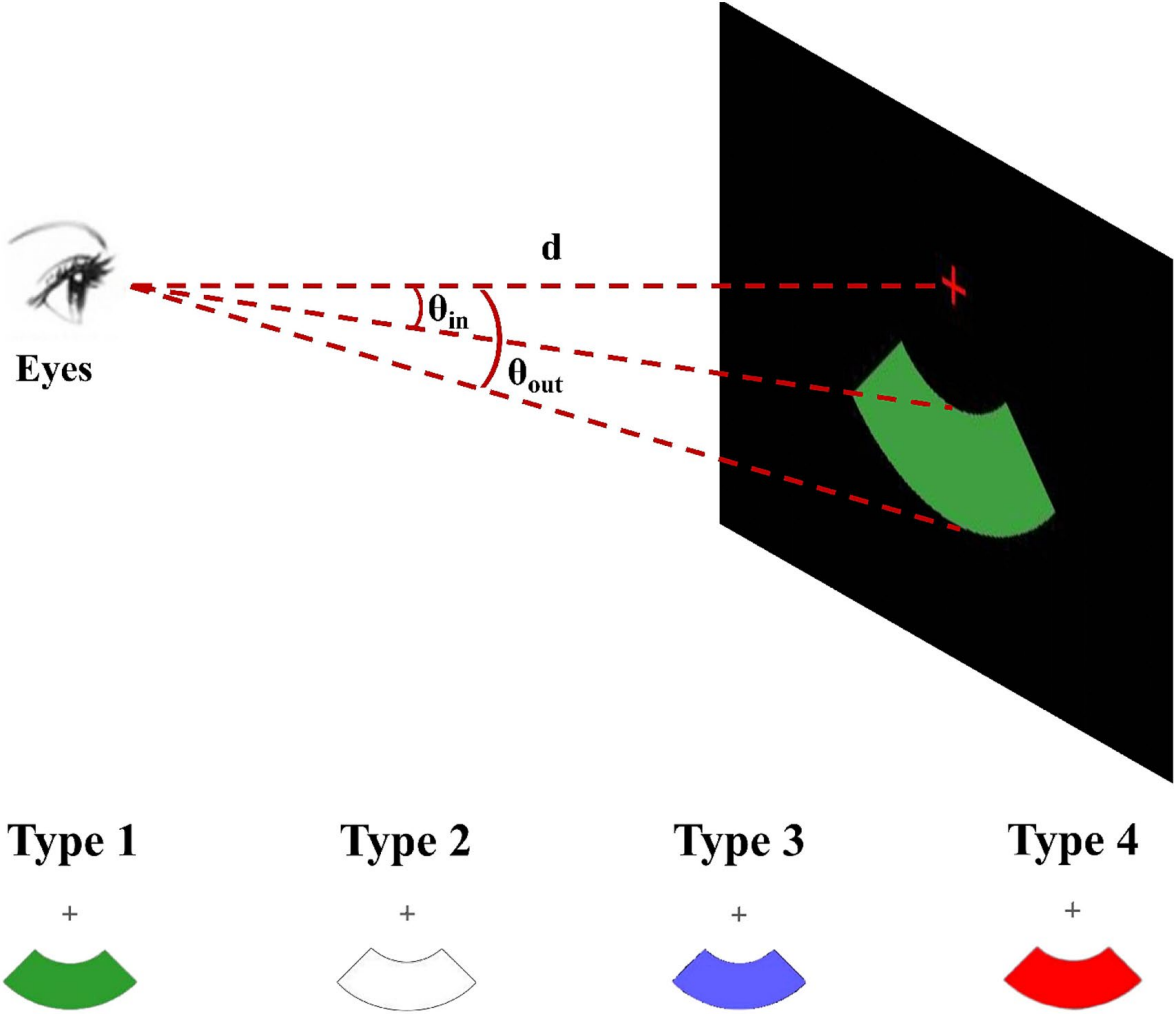
Visual stimulus design for this study. The visual attention point (red cross) is located at the center of the screen and at eye level, with a 50-cm vertical distance (d). The inner eccentricity of the visual stimulus fan pattern ( $\theta_{\text{in}}\;$ ) is set to 2.1°, and the outer eccentricity ( $\theta_{\text{out}}$ ) is set to 4.1°. And four types of color stimulation are used for the offline experiment.

This study used peripheral visual field stimulation to induce SSVEPs. As shown in Figure 1, an annular stimulation area was selected to present visual stimuli within a polar angle range of −135° to −45°. Participants were asked to fixate on the central cross during the presentation of the flickering stimulus. To enhance user comfort, this study adopted an ultra-low-frequency peripheral stimulation paradigm. The selection of the specific frequency range (2 Hz to 3.32 Hz with an interval of 0.12 Hz) was based on the optimized ultra-low frequency paradigm proposed by Pang et al. (2025), which was designed to balance user comfort and ITR.

This study employed color-modulated visual stimuli using three conventional colors of red, green and blue with white serving as the reference condition. The selection of these colors was based on the trichromatic theory of vision and their established use as benchmarks in BCI performance and comfort (Zhu et al., 2023; Reitelbach and Oyibo, 2024). Secondary colors (e.g., yellow and cyan) were excluded to avoid overlapping cone activation, thereby clarifying the unique contribution of each primary color to peripheral SSVEP modulation.

To minimize the potential confounding effect of stimulus luminance on SSVEP responses, a luminance-balancing strategy was employed for the stimuli. Rather than using raw RGB values, the HSV parameters were specifically adjusted to compensate for the human eye’s spectral sensitivity (Stockman, 2019). These parameters were determined via a subjective brightness-matching task to achieve uniform perceived intensity: red (hue: 0°, saturation: 100%, brightness: 100%), green (hue: 120°, saturation: 60%, brightness: 60%), and blue (hue: 240°, saturation: 60%, brightness: 100%). After the visual test, we verified the balance by calculating the relative luminance ( Y ) of each color (Equation 1). This calculation followed the ITU-R BT.709 standard (International Telecommunication Union, 2015) and the standard display gamma ( γ=2.2 ):


$$Y=0.2126\cdot R^{\gamma }+0.7152\cdot G^{\gamma }+0.0722\cdot B^{\gamma }$$ (1)

The calculated relative luminance values for the red, green, and blue stimuli were 0.213, 0.245, and 0.196, respectively (normalized to the white reference at 1.000). This analysis confirmed that the perceived intensities were successfully balanced within a narrow range, ensuring that the SSVEP signal characteristics were primarily influenced by the color differences and flicker frequencies rather than variations in physical intensity.

Stimuli were presented on a 1920 × 1,080 resolution monitor with a refresh rate of 120 Hz. The monitor was positioned at a distance (d) of 50 cm from the user. To ensure stable fixation, several measures were implemented. First, a headrest was used to stabilize participants’ chins and foreheads, significantly restricting involuntary head and eye movements. Second, all participants were explicitly instructed to maintain gaze on the central cue and were observed by the experimenter throughout the trials. Any trial with visible gaze shifts or head movements was excluded. The stimulation signals were generated by using sinusoidal modulation encoding to ensure smooth luminance transitions. The stimulus presentation program was developed using Psychophysics Toolbox for MATLAB (Brainard, 1997).

### 2.3 BCI task design

#### 2.3.1 Offline experiment

This offline experiment was designed to evaluate stimulus presentation parameters and identify the optimal visual color-coding paradigm for peripheral vision-based systems. Four color conditions (i.e., red, green, blue, and white) were examined in this experiment. For each participant, the four-color conditions were randomly presented. Each color paradigm involved 10 experimental blocks, and within a block, the target stimulus color remained constant until all 10 blocks for that color were completed. Each block comprised 12 trials with frequencies from 2 Hz to 3.32 Hz in 0.12 Hz intervals. In the offline experiment, each trial lasted 6 s including 0.5 s for cue phase, 5 s for visual stimulation and 0.5 s for gaze shifting time. During stimulation phases, participants were instructed to avoid blinking and maintain fixation on the central visual cue rather than on the flickering stimuli. After completing each block, participants were offered optional rest periods. They were also required to complete a subjective questionnaire to evaluate the comfort level of each color. Following the assessment methodology of Zhao et al. (2021), a 6-point scale was employed to measure visual comfort, with scores ranging from 1 (totally unacceptable) to 6 (have a good experience). The entire offline single-target experiment lasted approximately 60 min for each participant.

#### 2.3.2 Online experiment

**Figure 2:**
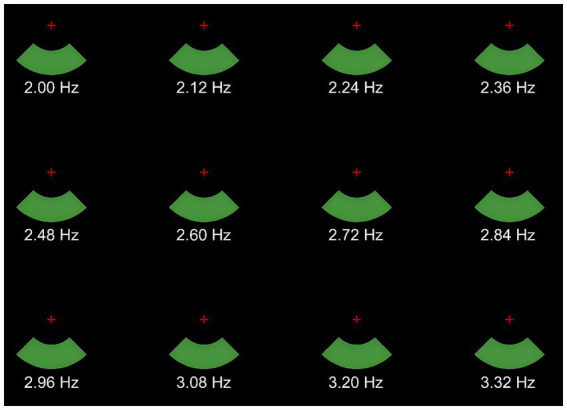
User interface of the proposed online 12-target SSVEP-based BCI system. The frequency corresponding to each target is marked below the visual stimulation.

According to the offline analysis results, green visual stimulation was adopted to build an online 12-target SSVEP-based BCI system to prioritize user comfort. Figure 2 shows the user interface of the proposed BCI system. Furthermore, a visual stimulation time of 3 s was used in the online BCI system. In the online experiment, each trial lasted 3.5 s including 3 s for visual stimulation and 0.5 s for gaze shifting time. The online experiment was divided into a training phase and a testing phase. There were 9 blocks in the training phase, and each block had 12 trials. The blocks in the training phase were used to derive SSVEP templates and filters for each participant. The testing phase included 10 blocks and each block contained 12 trials. Auditory feedback was provided to the participants in real time during the testing phase. A short beep was sounded after a target was correctly identified by the online data analysis program.

### 2.4 EEG recording

The study employed a SynAmps 2 system (Neuroscan, Inc.) for EEG acquisition at a sampling rate of 1,000 Hz. A 64-channel electrode cap, positioned according to the international 10–20 system, was used for signal collection. The reference electrode was located between Cz and CPz, and the ground electrode was positioned between Fz and FPz. Throughout signal recordings, electrode impedances were maintained below 10 kΩ. For the online experiment, nine electrodes (Pz, PO3, PO5, POz, PO4, PO6, O1, Oz, and O2) were selected for online recording. These electrodes are distributed over the visual cortex to ensure the effective acquisition of SSVEP signals (Liu et al., 2025). Synchronized trigger signals (marking stimulus onset) generated by the stimulation program were transmitted to the amplifier and logged in the event channel. Epoch extraction for subsequent analysis was performed based on these event markers.

### 2.5 Data processing

#### 2.5.1 Preprocessing and feature extraction

Data epochs were extracted based on event triggers from the stimulus program. Considering the visual system’s response latency (Ming et al., 2023a), the data epochs for offline and online experiments were extracted in [0.14 s 5.14 s] and [0.14 s 3.14 s] (time 0 indicated stimulus onset). All epochs were down-sampled to 250 Hz to reduce computational load and time cost. Multiple narrow band-pass infinite impulse response (IIR) filters were implemented for each participant. Fast Fourier transform (FFT) was performed to obtain frequency spectra. Signal-to-noise ratio (SNR), as defined in Equation 2, is calculated as the ratio of SSVEP amplitude spectrum to the average amplitude spectrum of the adjacent frequencies.


$$\mathrm{SNR}(f)=20\log_{10}\frac{10\times y(f)}{\sum_{k=1}^{4}[y(f-\mathrm{\Delta f}\times k)+y(f+\mathrm{\Delta f}\times k)]}$$ (2)

where y(f) is SSVEP amplitude spectrum at stimulation frequency f , Δf is equal to the reciprocal of the data length. During offline analysis, ocular artifacts were removed by using the EEGLAB toolbox and its ADJUST extension for MATLAB, which employed independent component analysis (ICA) to isolate artifact-related components. Automatically flagged artifacts were systematically excluded (Mognon et al., 2011; Pion-Tonachini et al., 2019).

#### 2.5.2 Classification algorithm

This study employed task-discriminant component analysis (TDCA) for SSVEP detection because it has been reported to have high classification performance (Liu et al., 2021). By projecting the EEG data into a subspace that maximizes between-class variance, while minimizing within-class variance, TDCA yields feature representations with the improved separability across stimulus categories.

The down-sampled and band-pass filtered EEG signal from trial i is denoted as $X_{i}$ . TDCA projects these signals onto the reference-signal subspace in order to produce an enhanced data matrix, from which the between-class scatter matrix $H_{b}$ (Equation 3) and within-class scatter matrix $H_{w}$ (Equation 4) are computed as


$$H_{b}=\frac{1}{\sqrt{N_{c}}}[{\bar{X}}_{a}^{1}-{\bar{X}}_{a}^{a},\ldots \ldots {\bar{X}}_{a}^{N_{c}}-{\bar{X}}_{a}^{a}]$$ (3)

$$H_{w}=\frac{1}{\sqrt{N_{t}}}[X_{a}^{\left(1\right)}-{\bar{X}}_{a}^{\left(1\right)},\ldots \ldots X_{a}^{\left(N_{t}\right)}-{\bar{X}}_{a}^{\left(N_{t}\right)}]$$ (4)

where $N_{c}$ is the number of classes, $N_{t}$ is the total number of trials, ${\bar{X}}^{i}$ and ${\bar{X}}^{\left(i\right)}$ denote the two-dimensional class centers of the i -th class and the i -th trial, respectively. The superscript a denotes all classes, and ${\bar{X}}_{a}^{a}$ is the global mean across all classes. The optimal spatial filter W (Equation 5) is then obtained by maximizing the Fisher criterion:


$$\max W=\frac{\mathrm{tr}(W^{T}S_{b}W)}{\mathrm{tr}(W^{T}S_{w}W)}$$ (5)

where $S_{b}$ and $S_{w}$ denote the between-class and within-class scatter matrices, derived from $H_{b}$ and $H_{w}$ . This projection matrix W is subsequently applied for feature extraction and classification.

#### 2.5.3 Performance evaluation

In this study, accuracy and ITR were calculated to evaluate the proposed system’s performance. The ITR was calculated using the following Equation 6 (McFarland et al., 2003):


$$\mathrm{ITR}=(\log_{2}N+P\log_{2}P+(1-P)\log_{2}(\frac{1-P}{N-1}))\times \frac{60}{T}$$ (6)

Where N denotes the number of targets, P represents the classification accuracy, and T represents the total trial duration, including the flicker stimulus duration and the gaze shifting time. In this study, a gaze shifting time of 0.5 s was included in the ITR calculation. Additionally, the comfort level of each experimental condition was assessed through a subjective questionnaire based on an existing study (Allison et al., 2010).

The subjective assessment was recorded on a 6-point scale, where 1 indicated ‘totally unacceptable’ and 6 represented ‘a good experience’. To systematically compare these measures, the one-way repeated measures ANOVA was conducted at a 5% significance level to investigate the effects of different color paradigms on both SSVEP responses and user comfort scores.

## 3 Results

### 3.1 Offline results

**Figure 3:**
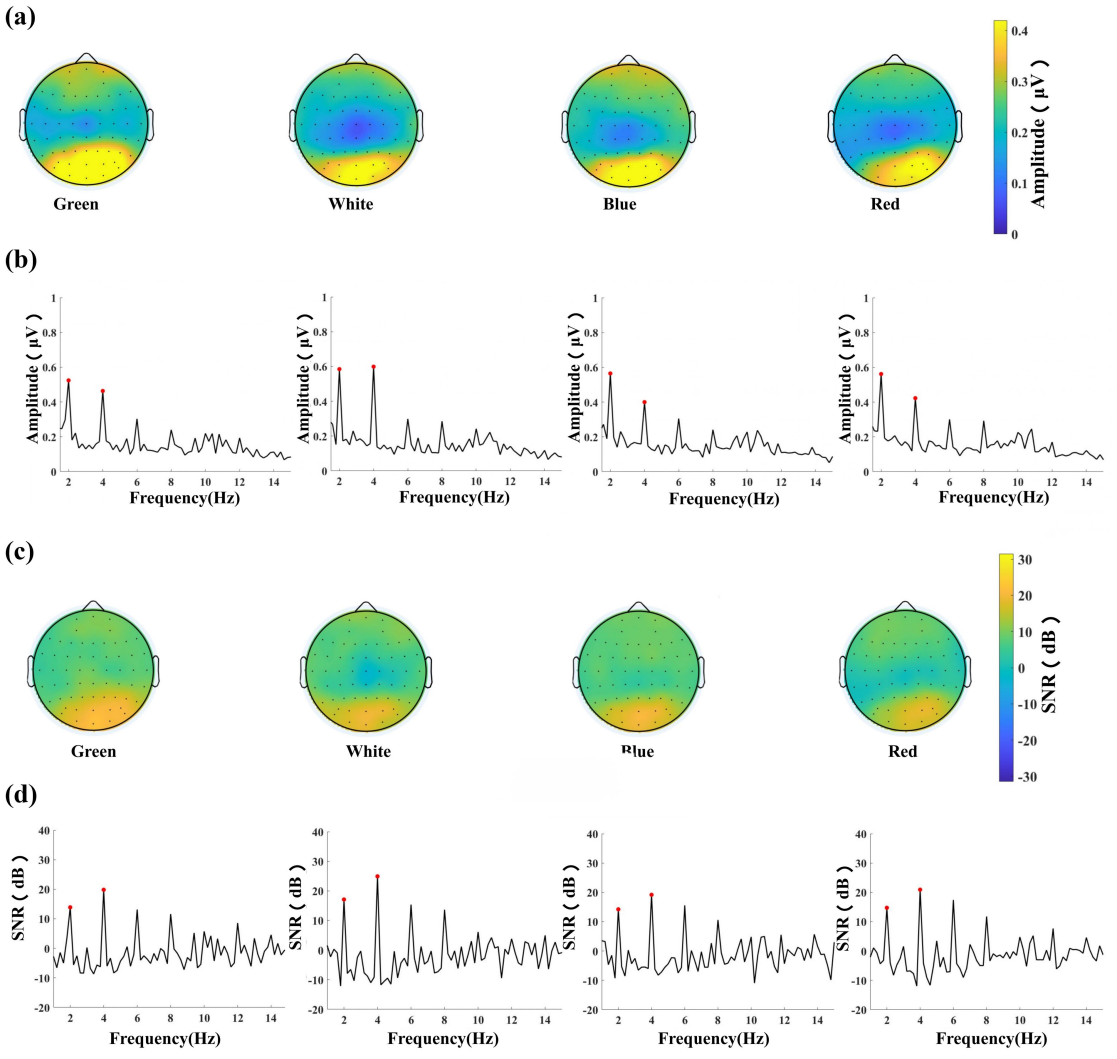
SSVEP responses averaged across all participants under four color stimulation paradigms at the stimulation frequency of 2 Hz. **(a)** Topographic maps of SSVEP amplitude. **(b)** Amplitude spectrum at the Oz electrode. **(c)** Topographic maps of SNR. **(d)** SNR spectrum at the Oz electrode.

Figure 3 illustrates the mean SSVEP responses averaged across all participants under four color stimulation paradigms. As shown in the topographic maps (Figures 3a,c), the SSVEP responses are primarily distributed in the occipital area. Given that consistent trends were observed across the nine occipital electrodes, Oz was selected as the representative channel for analysis due to its superior amplitude and SNR, consistent with previous SSVEP studies (Zheng et al., 2021). While multiple stimulation frequencies (ranging from 2 Hz to 3.32 Hz) were tested, we present the results for the 2 Hz condition as a representative example, as it clearly demonstrates the distinctive fundamental and harmonic peaks observed across all stimulation paradigms. These results indicate that peripheral visual stimulation at these frequencies can effectively and robustly induce stable SSVEPs in the visual cortex.

**Figure 4:**
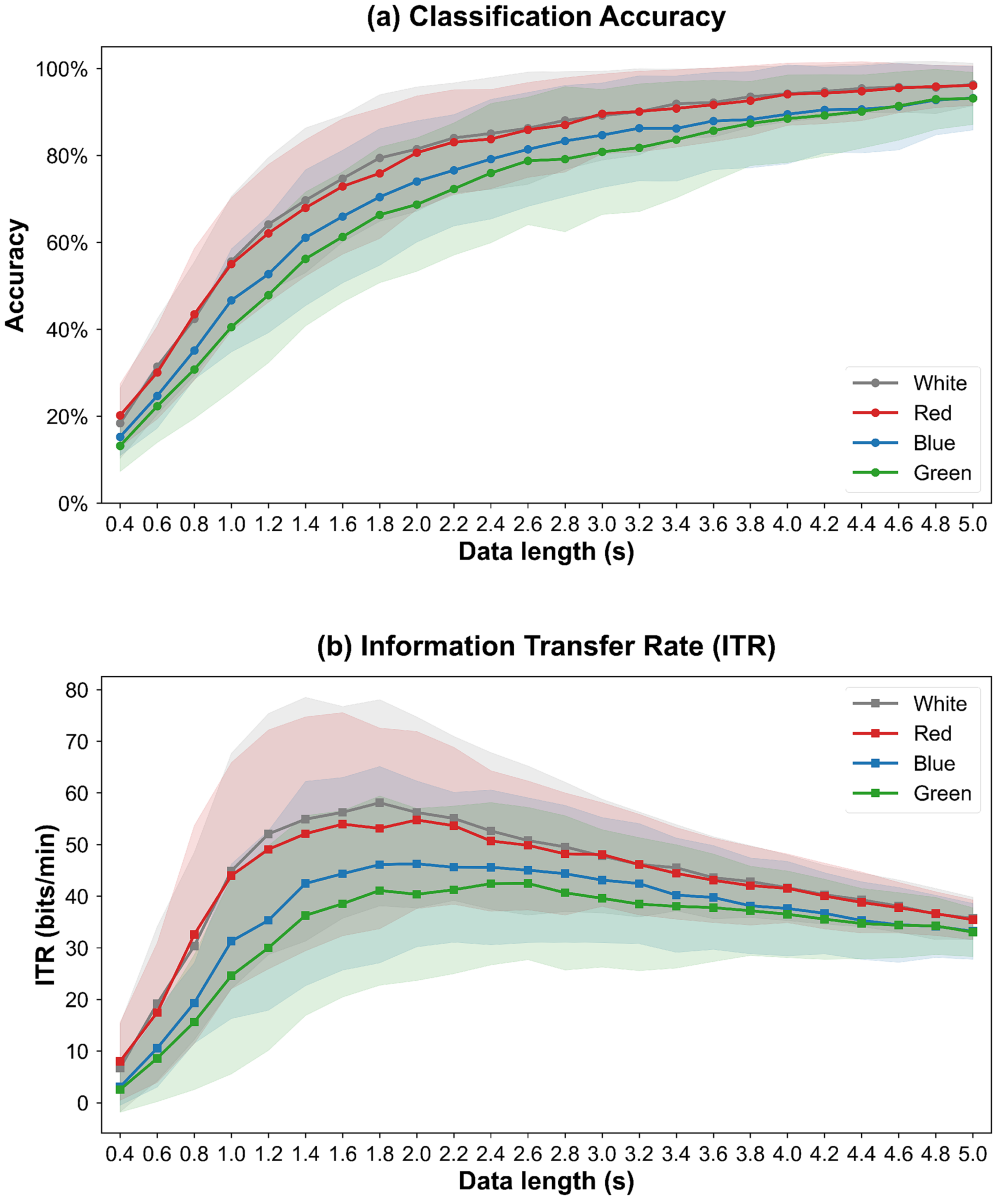
Mean classification accuracy **(a)** and ITRs **(b)** across participants as a function of data length. The shaded areas denote the standard deviation.

Figure 4 shows the average classification accuracy and ITR for the four stimulation colors, where the shaded areas represent the standard deviation. As shown in Figure 4, the classification accuracy increases with the increase of data length and then stabilizes. For each stimulation color, there is an inverted U-shaped relationship between ITR and data length. The white stimulus condition achieved the highest ITR (i.e., 58.09 ± 19.97 bits/min) when the data length was 1.8 s. The highest ITR was obtained when the data length was 2 s for both the red stimulus condition and the blue stimulus condition (i.e., 54.76 ± 17.17 bits/min and 46.26 ± 16.07 bits/min). The green stimulus condition achieved the highest ITR (i.e., 42.47 ± 14.75 bits/min) when the data length was 2.6 s. Among the four stimulus conditions, the highest ITR obtained with the white stimulus condition is the highest, and the highest ITR obtained with the green stimulus condition is the lowest. For each data length, one-way repeated measures ANOVA showed that there were no significant differences in ITR among four stimulus colors (*p* > 0.05 for all data lengths).

**Figure 5:**
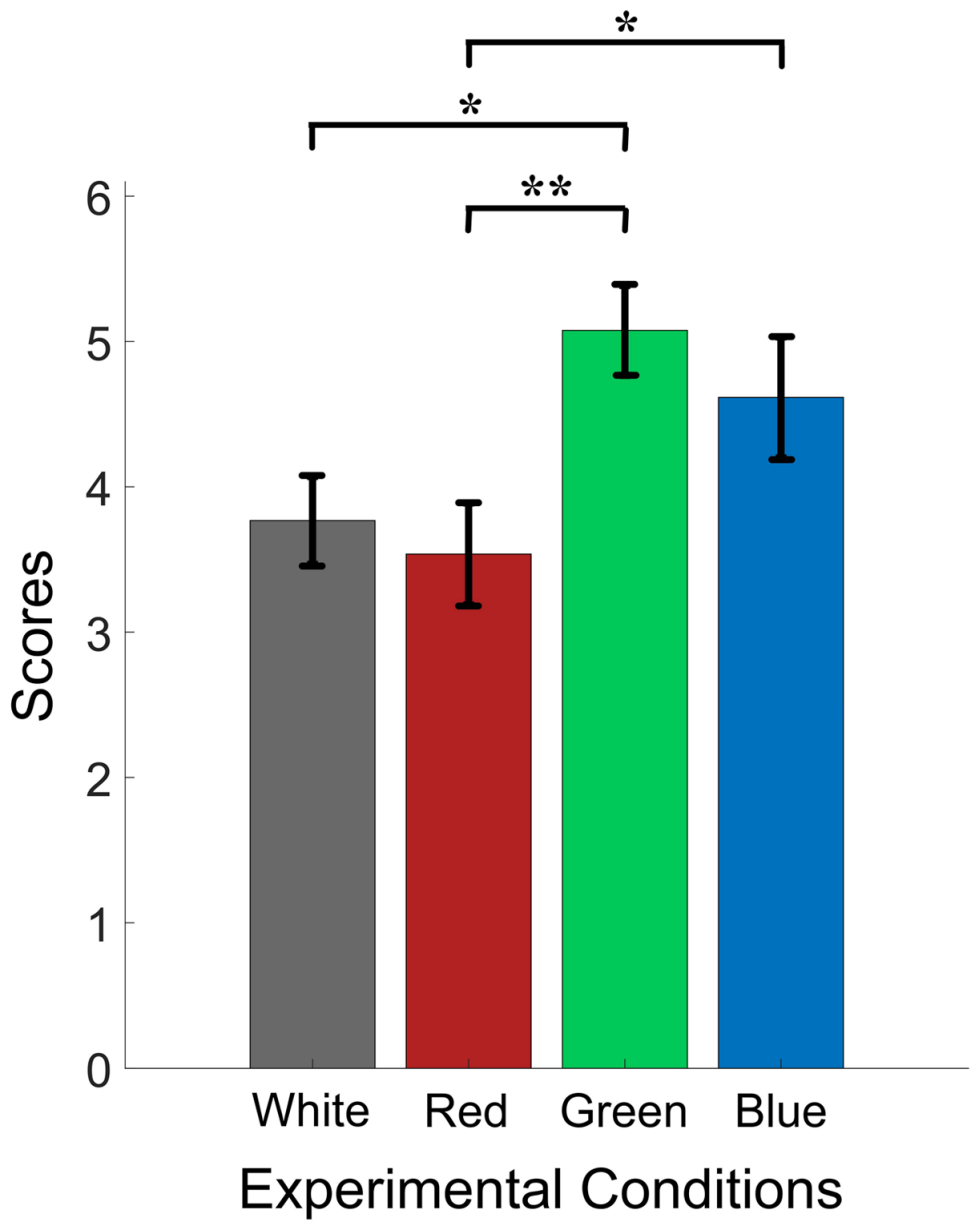
The outcomes of the subjective comfort questionnaire. The error bars indicate standard errors. Significant differences between pairs of color conditions were calculated using Fisher’s least significant difference *post hoc* test after one-way repeated measures ANOVA (*p < 0.05, **p < 0.01).

Figure 5 shows the results of the subjective comfort questionnaire. As shown in Figure 5, the green stimulus condition obtained the highest comfort score (5.08 ± 1.12) and the red stimulus condition achieved the lowest comfort score (3.54 ± 1.27). The effect of stimulus color on subjective comfort was analyzed using one-way repeated-measures ANOVA, followed by Fisher’s least significant difference (LSD) *post hoc* test for pairwise comparisons between color conditions. The results showed that green stimuli were rated as significantly more comfortable than both red and white stimuli, and blue stimuli were rated as significantly more comfortable than red stimuli. Since the green stimulus had the highest comfort score (see Figure 5), the green stimulus was adopted to build the online 12-target SSVEP-based BCI system. As shown in Figure 4 and Table 1, the green stimulus obtained the highest ITR at 2.6 s, with a corresponding accuracy was 78.78 ± 14.68%. When the data length extended to 3 s, the accuracy of green stimulus condition further improved to 80.83 ± 14.39%, and there was no significant difference between the ITR corresponding to the data length of 2.6 s and 3 s (*p* = 0.6057 > 0.05, one-way repeated measures ANOVA). Therefore, a visual stimulation time of 3 s was adopted in the online BCI system.

**Table 1:** Performance of the four stimulus colors at their respective peak ITR time points in the offline experiments.

Condition	ITR peak time (s)	Max ITR (bits/min)	Accuracy at ITR peak (%)	Comfort score
White	1.8	58.09 ± 19.97	79.42 ± 14.57	3.77 ± 1.09
Red	2.0	54.76 ± 17.17	80.64 ± 13.08	3.54 ± 1.27
Blue	2.0	46.26 ± 16.07	74.04 ± 13.97	4.62 ± 1.50
Green	2.6	42.47 ± 14.75	78.78 ± 14.68	5.08 ± 1.12

Data are presented as mean ± standard deviation (*n* = 13). The comfort score is based on a 6-point scale (1: totally unacceptable, 6: have a good experience).

### 3.2 Online BCI performance

According to the offline analysis results, green visual stimulation and 3-s visual stimulation time were adopted in the online experiment to prioritize user comfort. Table 2 lists the classification accuracy and ITR of the online experiment. As listed in Table 2, the online experiment achieved a mean classification accuracy of 89.93 ± 6.10% and an ITR of 47.96 ± 6.98 bits/min. The highest classification accuracy and the lowest classification accuracy were 98.33% and 80.00%, respectively. The high BCI performance verified the feasibility of the proposed 12-target SSVEP-based BCI.

**Table 2:** Classification accuracy and ITRs of the online BCI experiment.

Participants	Accuracy (%)	ITR (bits/min)
P1	80.95	38.12
P3	85.00	42.11
P4	93.33	51.45
P6	92.50	50.42
P7	97.50	57.08
P8	95.83	54.70
P10	90.00	47.49
P11	80.00	37.22
P13	90.83	48.44
P14	98.33	58.37
P15	85.00	42.11
Mean	89.93	47.96
± SD	6.10	6.98

## 4 Discussion

This study shows that stimulus color affected subjective comfort ratings while producing comparable BCI performance under the present peripheral ultra-low-frequency SSVEP setup. Our offline results confirmed that signal responses were centered in the occipital region, consistent with established spatial characteristics (Zhang et al., 2022). While classification accuracy remained stable across color conditions, green and blue stimuli were rated as significantly more comfortable. Based on these findings, our 12-target online system achieved an average accuracy of 89.93 ± 6.10% and an ITR of 47.96 ± 6.98 bits/min. This performance not only validates the feasibility of peripheral ultra-low frequency stimulation but also suggests a comfort-driven color choice.

The robustness of SSVEP responses in the lower peripheral field confirms that high BCI performance is achievable without foveal fixation (Zhang et al., 2019). Topographic analysis shows that primary activation remains concentrated in the occipital regions, mirroring central-field paradigms (Zhang et al., 2022). This spatial consistency validates standard electrode configurations, simplifying the transition to practical applications. By leveraging the high sensitivity of the lower retina (Hu et al., 2023) this approach maintains signal strength while reducing the interference and fatigue typical of central flickering—but the naturalness of this indirect attentional strategy still requires further validation.

In terms of color performance for peripheral vision, the four colors demonstrated no significant differences in classification accuracy and ITR. Participants gave green stimuli the highest comfort ratings among the tested colors, indicating better subjective visual tolerability in this sample. This comfort-related trend was consistent across both central and peripheral visual field paradigms (Zhu et al., 2023). Blue stimuli achieved comparable accuracy to other colors, and were more comfortable than red. Notably, this finding is distinct from prior SSVEP studies that reported weaker blue performance when compared with red, green, and white (Du and Zhao, 2022; Zhu et al., 2023). The improved performance of blue stimuli compared to central vision observations might be due to increased distribution of blue cone photoreceptors in peripheral vision (Liu et al., 2024). Consistent with our findings that red stimuli presented in the peripheral visual field received the lowest comfort scores, previous studies have also reported discomfort associated with red stimuli in the central visual field (Reitelbach and Oyibo, 2024). Previous studies also confirmed that red stimuli significantly increase arousal levels and elevate the risk of triggering photosensitive epileptic responses (Wilms and Oberfeld, 2018). Overall, the proposed BCI paradigm suggests a promising direction for developing more comfortable SSVEP-based BCI using peripheral visual stimulation.

A possible explanation for this color-related comfort pattern may involve the interaction of spectral sensitivity, perceived brightness, peripheral retinal processing, and cortical chromatic processing. Human cone photoreceptors show distinct wavelength-dependent spectral sensitivities, and photopic vision is highly sensitive to middle-wavelength light, which may allow green stimulation to remain perceptually salient without requiring excessive perceived intensity (Schnapf et al., 1987; Stockman, 2019). In addition, photoreceptor density and cone-opponent processing vary across retinal eccentricity, and red-green and blue-yellow opponent mechanisms show different distributions across the visual field (Curcio et al., 1990; Mullen and Kingdom, 2002). Therefore, color effects in peripheral SSVEP stimulation may not simply replicate those observed in central-field paradigms. At the cortical level, color information is transmitted through cone-opponent retinal and geniculate pathways and further integrated in visual cortex, where chromatic processing interacts with other visual attributes such as luminance, form, motion, and temporal modulation (Gegenfurtner and Kiper, 2003). Thus, the higher comfort rating of green stimulation in this study may reflect the combined influence of retinal and cortical visual-processing mechanisms rather than a single isolated factor. Because the present study did not use cone-isolating stimuli or objective physiological comfort measures, this interpretation should be regarded as a possible mechanistic account that requires further experimental verification.

Despite the promising results, several limitations of the current study should be acknowledged. First, the assessment of visual comfort relied solely on a subjective questionnaire, lacking objective physiological measures for visual fatigue or eye strain. Second, although participants were explicitly instructed to maintain central fixation, the absence of objective eye-tracking measures prevents the absolute exclusion of potential involuntary eye movements. Third, the current study was conducted with a relatively small sample size, and the participant demographic was limited to young, healthy adults (aged 18–25 years). To address these limitations and further optimize the system performance, future research will focus on the following aspects. We plan to increase the sample size and recruit a more diverse cohort across different age groups to validate these findings and explore broader applicability. Additionally, we will incorporate objective fatigue monitoring for a more comprehensive comfort evaluation. On the one hand, more efficient EEG classification algorithms will be employed to enhance the performance and robustness of the proposed system. Training-free methods and transfer-learning frameworks are particularly promising, as they have been successfully applied in code-modulated VEP (c-VEP) paradigms and may help reduce the current calibration burden (Martínez-Cagigal et al., 2021; Thielen et al., 2021). On the other hand, prior research has indicated that color performance is frequency-dependent, with significant differences between low and high frequency conditions (Duart et al., 2020). Furthermore, high-frequency stimulation offers superior visual comfort because it exceeds the critical flicker fusion threshold, rendering the flicker nearly imperceptible to the user. This characteristic provides a significant advantage in mitigating visual fatigue, which is a common limitation of lower-frequency stimuli. Therefore, we will explore the effects of color stimuli on peripheral SSVEP-based BCIs under high-frequency stimulation in future work. Finally, to move beyond the current constrained laboratory paradigm and rigorously control for eye movements, we plan to develop a hybrid SSVEP-eye-tracking BCI system. In this proposed framework, eye-tracking will be utilized for rapid target localization and objectively monitoring gaze behavior, while the SSVEP paradigm will serve as the reliable command execution mechanism. This hybrid approach will minimize prolonged exposure to flickering stimuli, thereby reducing visual fatigue and facilitating a more naturalistic and comfortable user interaction in real-world contexts.

## 5 Conclusion

In this study, we investigated the impact of stimulus color on peripheral ultra-low frequency SSVEPs. The offline results from 13 healthy individuals showed that while the four stimulus colors were comparable in terms of SSVEPs and classification performance, green visual stimulation received the highest subjective comfort score among the tested colors. Therefore, green visual stimulation was adopted to build an online 12-target peripheral ultra-low frequency SSVEP-based BCI. The online results achieved an average classification accuracy of 89.93 ± 6.10% and an average ITR of 47.96 ± 6.98 bits/min. These results provide preliminary evidence for the feasibility of the proposed BCI system using the group-level, comfort-driven green stimulus selection and enhanced our understanding of peripheral SSVEPs.

## Data Availability

The datasets presented in this article are not readily available because data and the programming code used as part of this research can be obtained from corresponding authors on reasonable request. Requests to access the datasets should be directed to chenxg@bme.cams.cn.
